# Cancer models, genomic instability and somatic cellular Darwinian evolution

**DOI:** 10.1186/1745-6150-5-19

**Published:** 2010-04-20

**Authors:** Mark P Little

**Affiliations:** 1 Department of Epidemiology and Biostatistics, School of Public Health, Imperial College Faculty of Medicine, Norfolk Place, London W2 1PG, UK; 2 Current address: Radiation Epidemiology Branch, National Cancer Institute, Executive Plaza South, 6120 Executive Boulevard MSC 7238, Bethesda, MD 20892-7238 USA

## Abstract

The biology of cancer is critically reviewed and evidence adduced that its development can be modelled as a somatic cellular Darwinian evolutionary process. The evidence for involvement of genomic instability (GI) is also reviewed. A variety of quasi-mechanistic models of carcinogenesis are reviewed, all based on this somatic Darwinian evolutionary hypothesis; in particular, the multi-stage model of Armitage and Doll (*Br. J. Cancer *1954:**8**;1-12), the two-mutation model of Moolgavkar, Venzon, and Knudson (MVK) (*Math. Biosci*. 1979:**47**;55-77), the generalized MVK model of Little (*Biometrics *1995:**51**;1278-1291) and various generalizations of these incorporating effects of GI (Little and Wright *Math. Biosci*. 2003:**183**;111-134; Little *et al. J. Theoret. Biol*. 2008:**254**;229-238).

**Reviewers:**

This article was reviewed by RA Gatenby and M Kimmel.

## Synopsis

The biology of cancer is reviewed and evidence adduced that it can be modelled as a somatic cellular Darwinian evolutionary process; evidence for involvement of genomic instability is also reviewed.

## Introduction

In this review article we shall critically review evidence on initiation and progression of cancer. In particular we shall attempt to justify why cancer can be treated as a somatic cellular Darwinian evolutionary process. A variety of quasi-mechanistic models of carcinogenesis will be reviewed, all based on this somatic Darwinian evolutionary hypothesis; in particular, the multi-stage model of Armitage and Doll [1], the two-mutation model of Moolgavkar, Venzon, and Knudson (MVK) [2,3], a multistage generalization of the MVK model of Little [4] and various generalizations of these incorporating effects of transmissible genomic instability (GI) [5,6]. In the "Biological background" section we shall review the basic biological data, and in the section "Genomic instability and somatic cellular Darwinian evolution in cancer" we shall examine the evidence for GI as an initiating event in cancer. In the section "Is somatic cellular Darwinian evolution in cancer plausible?" we shall consider the evidence for regarding development of cancer as a somatic Darwinian evolutionary process. Finally in the section "Carcinogenesis models and somatic cellular Darwinian evolution" we shall consider in turn various stochastic cancer models developed and widely employed in the last 50 years, all based on this hypothesis.

## Biological background

The biology of cancer is a vast subject and inevitably in a review of this nature one can only touch on what might be regarded as the more important and relevant themes - those needing more background biology are advised to consult one of number of basic texts, for example, the recent book by Weinberg [7].

Cancer is a group of diseases characterized by autonomous, uncontrolled cell proliferation, evasion of cell death, self-construction of oxygen and nutrient supply and spreading of cancerous cells through metastasis [7,8]. An early hypothesis postulated that the onset of cancers was a consequence of virus infections (see, for example, Stanley [9] for a review). Although many retroviruses and DNA viruses were identified in animal leukaemias and occasionally in human leukaemias [10-12], the vast majority of these 'cancer-related' viruses were not aetiologically involved in human cancers [10,12][7] (chapter 3) and only a few were direct carcinogens [13,14][7] (chapter 3). However, investigation of such viruses led to the discovery of the first human oncogene, *v-src*, whose nucleic acid sequences are similar to those of its viral homologue [15]. Together with the subsequent identification of tumour suppressor genes (TSG), the understanding of cancer origin has since been extended from external carcinogenic agents (i.e., retroviruses and chemical carcinogens) to alterations in the host genome [16,17][7] (chapter 11). The key tenet of the latter understanding is that cancer results from accumulation of changes to the DNA in somatic cells [18,18-20][7] (chapter 11). These data and others consistently identify modifications to key components in the somatic cell genome as responsible for initiating and sustaining the cancer process. We review this literature in the section "Genomic instability and somatic cellular Darwinian evolution in cancer" below.

Cells divide by duplicating their genetic material, a process termed the cell cycle. This consists of five distinct phases, G0 (quiescent), G1, S (synthesis), G2 (G1+S+G2 are collectively known as interphase) and M phase (mitosis). M phase is itself composed of two tightly coupled processes: mitosis, in which the cell's chromosomes are divided between the two daughter cells, and cytokinesis, in which the cell's cytoplasm divides forming distinct cells. Since integrity of the genome, and in particular chromosomes, is crucial in maintaining normal cell function, the cell cycle is closely monitored at various checkpoints [7] (chapter 8). In particular, the *APC *[21], *p53 *and *RB1 *[22,23] genes have been implicated in G1/S checkpoint control. Detection of DNA damage in cells may result in cell cycle arrest so that damage can in some cases be repaired [24,25][7] (chapter 8) or the damaged cells may undergo apoptosis [26,7] (chapter 8). In addition, during DNA segregation, the spindle assembly checkpoint ensures that all chromosomes are properly connected by the mitotic spindle [27,28].

DNA mutations occur randomly or as a result of exogenous mutagenic exposures. The majority of these mutations have little or no effect (e.g., silent mutations). Furthermore, depending on the nature of the damage, some can be repaired by specific DNA repair mechanisms. Base excision repair deals efficiently and accurately with single base damage, utilizing the intact complementary DNA strand as the template for repair [29][7] (chapter 12). On the other hand, double strand breaks (DSBs), resulting from cuts in both DNA strands, are more complex and potentially more detrimental. There are two major DSB repair mechanisms, namely non-homologous end joining (NHEJ) and homologous recombination (HR) [7] (chapter 12). NHEJ repairs the damage by simply merging the two ends of the break through DNA ligation. HR repairs the breaks either by using sequences in a homologous chromosome or a sister chromatid as the repair template or through single strand annealing (SSA) [7] (chapter 12). In the latter case the intervening region between two identical repeated sequences residing on either side of the DSB is removed and the two repeated sequences are merged. In each case, HR requires the presence of homologous DNA sequences, which reduces the potential errors in repair. In contrast, because of the lack of a complementary repairing template, NHEJ is particularly error-prone [29][7] (chapter 12). Mis-ligation of the two ends resulted from NHEJ, for example, is implicated in chromosome translocations in acute lymphoid leukaemia [30].

Whether induced by exogenous or endogenous mutagens or introduced during reconstruction of the damaged DNA, either a single base pair can be modified or there can be a larger-scale event such as gain or loss of a chromosome segment. A mis-sense mutation replaces the original amino acid with a different one while a nonsense mutation shortens the affected protein sequence and ultimately leads to protein degradation. Due to the absence of a particular protein or a group of proteins, mis-sense and nonsense mutations are often lethal to the affected cell. In addition, insertion or deletion of base pairs can lead to frameshift mutations, which may completely change the protein sequence.

Chromosomal abnormalities, that is to say large scale alterations to the DNA, be they deletions, duplications or translocations, can have more severe effects. Chromosome translocations occur when a stretch of DNA is moved from its original chromosomal position to another position and may result from mis-repair of DSBs and mutations in DNA-repair pathways [31]. Specific chromosome translocations are observed in both acute myeloid leukaemia, in which positions q22 on both chromosomes 8 and 21 are frequently exchanged [32], and chronic myeloid leukaemia, characterized by the presence of the *BCR-ABL *hybrid gene that increases the rate of division and evades apoptosis [33]. Such abnormalities can result in amplification of a chromosome region and consequent over-production of relevant protein; deletion and loss of heterozygosity (LOH) will lead to loss of one or both copies of certain genes and their products. Deletion of the chromosome regions containing the *BRCA1 *and *BRCA2 *genes, for example, are commonly observed in inherited ovarian cancer and breast cancer [34,35] and complete inactivation of the *APC *gene, a tumour suppressor gene related to a number of cancers, is caused by LOH in oesophageal and non-small cell lung cancer [36,37] and other specific cancer types [7] (chapter 7).

When a mutation changes a gene in the germ line cells, it may be passed on to offspring, whose component cells, as a result, all contain a defective copy of the gene. For example, compared with children who are born with a normal, intact *RB1 *gene, those born with a germinal mutation in one of the two *RB1 *alleles have an enhanced risk of developing retinoblastoma (RB), a childhood ocular malignancy [38,39]. Furthermore, in contrast to the sporadic (homozygous) cases, over 60% of the inherited RB cases are bilateral, i.e., tumours appear in both eyes [38]. Although germ-line mutations are relatively rare, the inherited defects exhibited in all cells in the body predispose the heterozygous individual to various genetic disorders, including cancers.

Mutations to somatic cells, like their germinal counterparts, may cause diseases in the host organ. As indicated above, there are two main classes of genes directly involved in carcinogenesis, oncogenes and TSGs [27][7] (chapters 4, 7). Activation of an oncogene requires only a single mutation to one of the two homologous alleles of a proto-oncogene; the remaining intact allele cannot compensate for the resulting dominant oncogenic defect. In contrast, TSGs are recessive, i.e., one wild-type allele of the gene can maintain normal function. Complete inactivation of the growth suppression function from TSGs, as for example in RB, therefore, requires two mutations.

Immortality is a distinctive characteristic of cancer cells. It is known that normal somatic cells can only divide up to a limited number of times (the Hayflick limit) and once this limit is reached, they enter replicative senescence and lose the ability to divide further [40,41][7] (chapter 10). Telomere shortening is a possible mechanism implicated in limiting a cell's division potential [41]. In humans, the telomere is a sequence of several thousand repeats (TTAGGG) residing at the end of every chromosome. Its existence prevents the loss of vital genetic information at each end of the chromosomes and protects genomic integrity by inhibiting chromosomal fusions (joining of two chromosomes) [42][7] (chapter 10). The loss of a certain length of the telomere after each cell division gradually diminishes the cell's division potential and ultimately leads to cell senescence or death [43][7] (chapter 10). By contrast, telomeres in most cancer cells remain above the critical length so that the restriction on division number imposed by telomere shortening is lifted and hence cancer cells can multiply without limit [44][7] (chapter 10). One mechanism in cancer cells to counteract telomeric shortening is activation of telomerase, an enzyme that maintains the length by adding the hexanucleotide onto the end of the telomere [45,46][7] (chapter 10). Although 85-90% of tumour cells express telomerase, a certain proportion of such cells do not [47][7] (chapter 10); the precise mechanisms by which these cells maintain telomere length are unclear, although an interchromosomal copying mechanism is implicated [48][7] (chapter 10).

When a cell has acquired the malignant phenotype, classically it is assumed to multiply quickly to a clinically overt tumour. However, like normal tissues, tumours require an adequate supply of oxygen, metabolites and an effective way to remove waste products [49,7] (chapter 13). However, these requirements vary among tumour types, and change over the course of tumour progression [50]. Gaining access to the host vascular system and the generation of a tumour blood supply are rate-limiting steps in tumour progression, and require what has been termed an "angiogenic switch" [51][7] (chapter 13). The interaction of the tumour with the microvasculature is discussed in a bit more detail below.

## Genomic instability and somatic cellular Darwinian evolution in cancer

As cells acquire subsequent mutations, they acquire selective advantage over cells not having these mutations, manifest in a loss of cell cycle control, lack of response to external signals and ultimately higher rates of cell turnover. As such this corresponds to a process that might be termed "somatic Darwinian evolution" [52,53]. Vineis and Berwick [54] present a variety of evidence that suggests that the somatic development of cancers in populations arise as a result of selective pressures induced by a variety of environmental stimuli. Gatenby *et al*. [55] and Smallbone *et al*. [56] have constructed cancer models allowing for precisely this feature, as we discuss in the sub-section "Malignant cell growth and clonal extinction". We discuss this critical assumption in more detail in the section "Is somatic cellular Darwinian evolution in cancer plausible?" below.

The classical view is that the cellular "mutations" are genetic or possibly epigenetic events that are clonally expressed in all cells and their descendents. Consistent with this, and as outlined by Harris [57] (but see also UNSCEAR [58]), there is compelling biological data to suggest that cancer arises from a failure of cell differentiation, and that it is largely unicellular in origin. There is also a large body of data, which does not necessarily contradict this hypothesis, showing the importance of the micro-environment in initiating and modifying tumour growth, indeed in tumour reversion, at least for certain tumour types (e.g., breast cancer) [59-66]. This has been termed the "field" theory. As discussed above, tumour growth requires additional vascular growth, the so-called "angiogenic switch" [51] [7] (chapter 13), without which it will not grow or metastasize. However, the importance of the micro-environment for the induction (rather than progression) of a large number of types of cancer has been disputed, since for many tumours there is clear evidence of clonality [57,58,63,67]. There is biological data suggesting that the initiating lesion in the multistage process leading to cancer might be one involving a destabilization of the genome resulting in elevation of mutation rates, reviewed by Morgan [68,69]. This might result from inactivation of one or more "caretaker" genes, responsible for maintaining genomic integrity [70], as opposed to the "gatekeeper" TSGs and proto-oncogenes discussed above. This destabilization would be expected to result in non-clonal expression of various mutations. Loeb [71,72] has presented evidence that an early step in carcinogenesis is mutation in a gene controlling genome stability. Stoler *et al*. [73] showed that there are 11,000 mutations per carcinoma cell for a number of different cancer types, again implying that genomic destabilization is an early event in carcinogenesis. In particular, there is data to suggest existence of such an early genomic destabilization event for colon cancer [71-73].

There is known to be heterogeneity in the types of GI that occur, particularly for colon cancer. The majority of human cancers exhibit chromosomal instability (CIN), characterized by cells having a large number of acquired abnormalities at the chromosomal level, expressed as gain or loss of large chromosome fragments, changes in chromosome number [74,75] and LOH [27]. A large proportion of colon cancers express loss of chromosome arms, often containing specific tumour suppressor genes such as *p53* (17p), *SMAD4 *and *APC *(5q) [18]. However, about 17% of colon cancers [76], as well as a generally smaller portion of other solid cancers [77], exhibit microsatellite instability (MIN), a less prevalent form of GI. MIN is caused by defects in the mismatch repair (MMR) mechanism, which contributes to replication fidelity by correcting incorrectly inserted DNA bases [27][7] (chapter 12). Defects in the MMR pathway lead to frequent insertions and deletions of repetitive short sequences, so-called microsatellites, across the genome. Several genes involved in MMR have been discovered in humans, for example, the *hMSH2 *gene on chromosome 2p16 [78,79] and the *hMLH1 *gene on chromosome 3p21-23 [80,81]. MIN is predominantly associated with hereditary non-polyposis colorectal cancer (HNPCC), but is not often seen in sporadic cases. In most HNPCC cases, patients exhibit cells that contain one mutant allele of the *hMSH2 *gene, inherited from either the paternal or maternal carrier, and one normal allele [78,79]. The existence of the wild-type allele acts dominantly, maintaining the mismatch repair function. If a sporadic mutation inactivates the remaining normal allele, the cell expresses the MIN phenotype, which results in an enhanced microsatellite and point mutation rate [27]. However, cancers from HNPCC patients are generally chromosomally normal, while MMR proficient tumours are generally aneuploid and highly chromosomally unstable [27]. Breivik [82,83] presents evidence that GI arises as a result of selection of cells in relation to specific mutagens in the environment; in particular he argues that the tissue specificity of CIN and MIN within the colon may result from adaptive selection associated with exposure to different agents, for which there is experimental support [84]. Chow and Rubin [85] demonstrate that cell selection is sufficient to explain the apparently increased mutation rates observed in cloned cell sub-populations *in vitro *- the assumption of GI is not required.

However, the question of whether chromosomal instability is the initiating event in carcinogenesis, even in relation to colon cancer (where the evidence is strongest), is controversial. Tomlinson *et al*. [86] point out that conventional mutation rates are entirely adequate to account for the observed incidence of colon cancer. Tomlinson and Bodmer [87] argue that cancer is an evolutionary process, and that the observed accumulation of chromosomal and other damage in colon cancers may simply be the result of selection for cells with growth advantage, with mutations "piggy-backing" on this process of selection. As above, Chow and Rubin [85] present experimental *in vitro *evidence that also suggests that GI is not necessary to induce neoplastic transformation - selection is sufficient. Much other evidence on the importance of cell selection for carcinogenesis is reviewed by Rubin [88]. As shown by Little and Li [89] and Little *et al*. [6] (and as we discuss in the sub-section "Multiple pathway models incorporating genomic instability" below), the fact that the two cancer stage GI model developed by Little and Wright [5] and similar models allowing for multiple types of GI [6], as well as the GI model of Nowak *et al*. [90] fit US Surveillance, Epidemiology and End Results (SEER) colon cancer data as well as, but no better than, the non-GI model of Luebeck and Moolgavkar [91] suggests that, based on the fit of these models to this population-based data, there is little evidence for or against the involvement of GI in colon cancer.

## Is somatic cellular Darwinian evolution in cancer plausible?

A common assumption of most carcinogenesis models, in particular all those discussed in the section "Carcinogenesis models and somatic cellular Darwinian evolution" below, is that all cell populations are independent, corresponding to the assumed somatic cellular Darwinian evolution. More rigorously, in mathematical terms we assume that cells with variable numbers of acquired mutations are statistically conditionally independent (conditional on the parental lineage and exogenous exposures), so that the cell populations may be described by a branching process. This is assumed for analytic tractability, but it is difficult to test.

To the extent that it is known that normal cells communicate with each other via cell surface markers and otherwise, this appears unlikely to be precisely true. One tissue in which, because of its spatial structure, this assumption may appear to break down is the colon. The colon and small intestine are structured into crypts, each crypt containing some thousands of cells, and organized so that the stem cells are at the bottom of the crypt [92,93]. There is evidence that there may be more than one stem cell at the bottom of each crypt [94]. The progeny of stem cells migrate up the crypt and continue to divide, becoming progressively more differentiated. The differentiated cells eventually reach the top of the crypt where they are shed into the intestinal lumen. Potten and Loeffler [92] and Nowak and colleagues [93,95] have postulated similar models for cancers of the small intestine and colon taking account of the linear structure of the crypts, and in which necessarily the assumption of conditional independence breaks down.

However, if mutation is regarded at the level of the crypt, then conditional independence of cell lineages is still likely to be true. Moreover, there is abundant evidence that, in contrast to normal cells, which rely on mitogenic stimuli, e.g., via TGF*β*, for proliferation, cancer cells do not depend on such external signals, in particular TGF*β*, for sustained growth, and are self-sufficient in this respect [96,97][7] (chapter 5). There is also data to suggest that inactivation of TGF*β *signalling is an early event in pancreatic cancer [98]. To this extent, tumour and pre-malignant transformed cells are likely to operate independently of cells in the vicinity, so that for these cells (the ones of critical importance in the models discussed above) the hypothesis of conditional statistical independence is not implausible.

However, statistical independence is unlikely to apply in late-stage processes, for example in the growth of the malignant cell clone, where there is very likely to be modulation of cell turnover and necrosis as the tumour size increases, especially if the angiogenic switch is not activated.

## Carcinogenesis models and somatic cellular Darwinian evolution

In this section we shall treat the major carcinogenesis models developed and used over the last 50 years. These and other models are discussed at greater length by Little [99].

### Armitage-Doll multistage model

**Figure 1:**
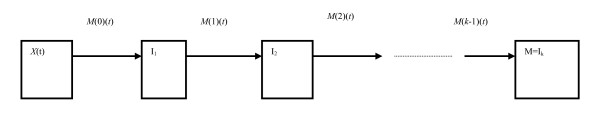
**Schematic diagram of the Armitage-Doll **[1]**multi-stage model**.

**Figure 2:**
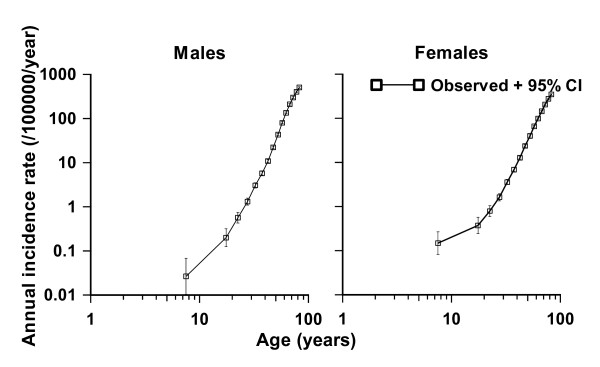
**SEER 1973-1999 **[164]**colon cancer data, and observed data (with 95% confidence intervals (CI), adjusted for overdispersion **[165]), **taken from Little **[99]. The use of double logarithmic (log-log) axes shows that except for the youngest age group (<10 years) the age-incidence relationship is well described by *C*·[age]^*k*-1^.

One of the more commonly observed patterns in the age-incidence curves for epithelial cancers is that the cancer incidence rate varies approximately as *C*·[age]^*β *^for some constants *C *and *β *[100,101]. The so-called multi-stage model of carcinogenesis of Armitage and Doll [1] was developed in part as a way of accounting for this approximately log-log variation of cancer incidence with age. The model supposes that at age *t *an individual has a population of *X(t) *completely normal (stem) cells and that these cells acquire one mutation at a rate *M*(0)(*t*). The cells with one mutation acquire a second mutation at a rate *M*(1)(*t*), and so on until at the (*k*-1) th stage the cells with (*k*-1) mutations proceed at a rate *M*(*k*-1)(*t*) to become fully malignant. The model is illustrated schematically in Figure 1. It can be shown that when *X*(*t*) and the *M*(*i*)(*t*) are constant, a model with *k *stages predicts a cancer incidence rate that is approximately given by the expression *C*·[age]^*k*-1 ^with *C *= *X·M*(0)·*M*(1)·...·*M*(*k*-1)/(1·2·...·(*k*-1)) [1,102]. As can be seen from Figure 2, for colon cancer the age-incidence relationship is remarkably well described by a power of age, as predicted by this model.

Departures from this form of relationship are only apparent at very young ages (< 10 years) (Figure 2). For many common epithelial cancers in adulthood this function, *C*·[age]^*k*-1^, fits the age-incidence and age-mortality relationships well, with the implied number of rate-limiting stages, *k*, between 5 and 7 [101]. In the intervening fifty years, there has accumulated substantial biological evidence (as reviewed in the sections "Biological background", "Genomic instability and somatic cellular Darwinian evolution in cancer", "Is somatic cellular Darwinian evolution in cancer plausible") that cancer is a multi-step process involving the accumulation of a number of genetic and epigenetic changes in a clonal population of cells.

However, there are certain problems with the model proposed by Armitage and Doll [1] associated with the fact that, as noted above, to account for the observed age incidence curve *C*·[age]^*β*^, between 5 and 7 rate-limiting stages are needed. This large number of stages implies high mutation rates in order to account for the observed number of cancers. Moolgavkar and Luebeck [103] fitted the Armitage-Doll multi-stage model to datasets describing the incidence of colon cancer in a general population and in patients with familial adenomatous polyposis. Moolgavkar and Luebeck [103] found that Armitage-Doll models with five or six stages gave good fits to these datasets, but that both of these models implied mutation rates that were too high by at least two orders of magnitude compared with experimentally derived rates. The discrepancy between the predicted and experimentally measured mutation rates might be eliminated, or at least significantly reduced, if account were to be taken of the fact that the experimental mutation rates are locus-specific. A "mutation" in the sense in which it is defined in this model might result from the "failure" of any one of a number of independent loci, so that the "mutation" rate would be the sum of the failure rates at each individual locus.

Notwithstanding these problems, much use has been made of the Armitage-Doll multi-stage model as a framework for understanding the time course of carcinogenesis, particularly for the interaction of different carcinogens [104].

### Two-mutation model

In order to reduce the arguably biologically implausibly large number of stages required by their first model, Armitage and Doll [105] developed a further model of carcinogenesis, which postulated a two-stage probabilistic process whereby a cell following an initial transformation into a pre-neoplastic state (initiation) was subject to a period of accelerated (exponential) growth. At some point in this exponential growth a cell from this expanding population might undergo a second transformation (promotion) leading quickly and directly to the development of a neoplasm. Like their previous model, it satisfactorily explained the incidence of cancer in adults, but was less successful in describing the pattern of certain childhood cancers.

**Figure 3:**
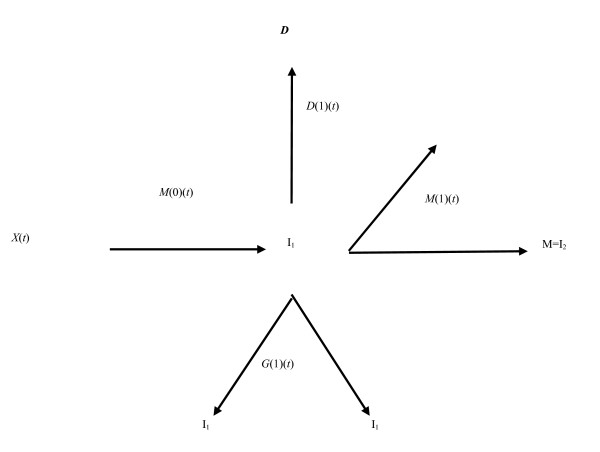
**Schematic diagram of the two-mutation (MVK) model **[2].

The two-mutation model developed by Knudson [3] to explain the incidence of retinoblastoma in children took account of the process of growth and differentiation in normal tissues. Subsequently, the stochastic two-mutation model of Moolgavkar and Venzon [2] generalized Knudson's model, by taking account of cell mortality at all stages as well as allowing for differential growth of intermediate cells. The two-stage model developed by Tucker [106] is very similar to the model of Moolgavkar and Venzon but does not take account of the differential growth of intermediate cells. The two-mutation model of Moolgavkar, Venzon and Knudson (MVK) supposes that at age *t *there are *X*(*t*) susceptible stem cells, each subject to mutation to an intermediate type of cell at a rate *M*(0)(*t*). The intermediate cells divide at a rate *G*(1)(*t*); at a rate *D*(1)(*t*) they die or differentiate; at a rate *M*(1)(*t*) they are transformed into malignant cells. The model is illustrated schematically in Figure 3. In contrast with the case of the (first) Armitage-Doll model, there is a considerable body of experimental biological data supporting this initiation-promotion type of model (see, e.g., [107,108]).

The model has been developed to allow for time-varying parameters at the first stage of mutation [109]. A further slight generalization of this model (to account for time varying parameters at the second stage of mutation) was presented by Little and Charles [110], who also demonstrated that the excess relative risk predicted by the model, when the first mutation rate was subject to instantaneous perturbation, decayed at least exponentially for a sufficiently long time after the perturbation. The model has been used by Moolgavkar *et al*. [111] and Heidenreich *et al*. [112,113] and many others to describe the incidence of lung cancer in rats exposed to radon, and in particular to model the inverse dose-rate effect that has been observed in this data. Moolgavkar *et al*. [114], Luebeck *et al*. [115], Hazelton *et al*. [116], Little *et al*. [117], Heidenreich *et al*. [118] and others have applied the model to describe the interaction of radon, smoking and other agents causing lung cancer in various miner cohorts. The two-mutation model has also been utilised to describe lung, stomach, and colon cancer in the Japanese atomic bomb survivor incidence data [119], and to fit to liver cancer data from a cohort of Swedish Thorotrast-exposed patients [120].

**Figure 4:**
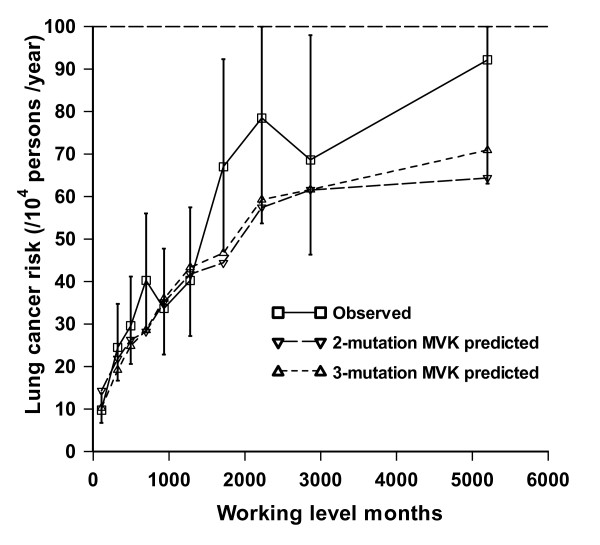
**Observed absolute risk of lung cancer mortality (+95% CI) and predicted risk associated with the optimal two-mutation and three-mutation models fitted to the Colorado Plateau uranium miner data as a function of cumulative radon-daughter exposure, taken from Little *et al*. **[117]

A curious finding in many analyses of lung cancer in relation to radon-daughter exposure using the two-mutation model is that there is significant radon action on intermediate cell proliferation. This has been observed in radon-exposed rats [112,113], in the Colorado Plateau uranium miners [115,117] and in the Chinese tin miners [116]. This is very much associated with fits of the two-mutation model, and may reflect the limited number of parameters that can be modified in this model. Analyses of rat data using a three-mutation generalized MVK model (see the sub-section "Generalized MVK and multistage models" below) did not find any indications of an effect of radon daughter exposure on intermediate cell proliferation [113]. Likewise, analysis of the Colorado Plateau miners using a three-mutation generalized MVK model (see the sub-section "Generalized MVK and multistage models" below) did not find any effect of radon daughter exposure on intermediate cell proliferation rates [117], and the fit of the three-mutation model was somewhat better than that of the two-mutation model (see Figure 4).

Moolgavkar and Luebeck [103] have used models with two or three mutations to describe the incidence of colon cancer in a general population and in patients with familial adenomatous polyposis. They found that both models gave good fits to both datasets, but that the model with two mutations implied mutation rates that were biologically implausibly low, by at least two orders of magnitude. The three-mutation model, which predicted mutation rates more in line with biological data, was therefore somewhat preferable. The problem of implausibly low mutation rates implied by the two-mutation model is not specific to the case of colon cancer, and is discussed at greater length by Den Otter *et al*. [121] and Derkinderen *et al*. [122], who argue that for most cancer sites a model with more than two stages is required. A possible way round the problem of implausibly low mutation rates, at least for colon cancer, is suggested by the model of Nowak *et al*. [93], who showed that by "washing out" pre-malignant cells in the intestinal lumen a relatively high mutation rate at the cellular level may translate into a much lower apparent mutation rate at the tissue (intestinal crypt) level.

Another problem with the two-mutation model is that when any of the model parameters are modified, there are relatively large fluctuations in the hazard function for carcinogenesis, which start almost as soon as the parameters are changed [4]. Moolgavkar *et al*. [114] partially overcome the problem posed by this instantaneous rise in the hazard after perturbation of the two-mutation model parameters in their analysis of the Colorado uranium miners data by assuming a fixed period (3.5 years) between the appearance of the first malignant cell and the clinical detection of malignancy. However, the use of such a fixed latent period only translates a few years into the future the sudden step-change in the hazard. To achieve the observed gradual increase in risk shortly after exposure, a stochastic process must be used to model the transition from the first malignant cell to detectable cancer, such as is provided by the final stage(s) in the three- or four-mutation generalized MVK models used in the analysis of Little [123] of the Japanese atomic bomb survivor data. In particular, an exponentially growing population of malignant cells could be modelled by a penultimate stage with *G*(*k*-1) > 0 and *D*(*k*-1) = 0, the probability of detection of the clone being determined by *M*(*k*-1). Alternatively, to allow for possible stochastic extinction of malignant clones (e.g., as a result of failure of the angiogenic switch) one could have a birth-death process, allowing both *G*(*k*-1) > 0 and *D*(*k*-1) > 0. Tan [124] has constructed an explicit model of such a process with time-varying *G*(*k*-1)(*t*) and *D*(*k*-1)(*t*). In their analysis of lung, stomach and colon cancer in the Japanese atomic bomb survivor incidence data Kai *et al*. [119] did not assume any such period of latency, perhaps because of the long period after the bombings (12.4 years) before solid cancer incidence follow-up began in the Life Span Study (LSS). There are other ways in which an observed gradual increase in tumour risk after parameter perturbation could be achieved, in particular by assuming a random tumour growth rate, or by using a quantal response rate, relating probability of tumour detection to size, as outlined by Bartoszyński *et al*. [125].

### Generalized MVK and multistage models

**Figure 5:**
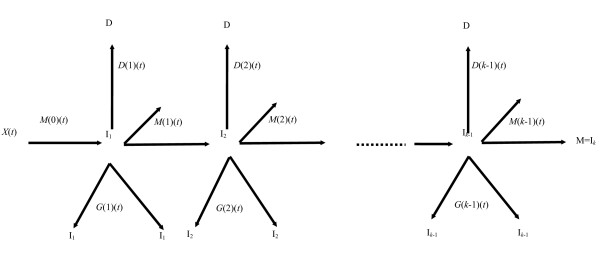
**Schematic diagram of the generalized MVK model **[4].

A number of generalizations of the Armitage-Doll and two- and three-mutation models have been developed [4-6,108]. In particular, two closely related models have been developed, whose properties have been described in the paper of Little [4]. The models generalize the two-mutation model of Moolgavkar, Venzon, and Knudson, and also the Armitage-Doll model, and will be termed the ***generalized MVK model***. For the generalized MVK model it may be supposed that at age *t *there are *X(t) *susceptible stem cells, each subject to mutation to a type of cell carrying an irreversible mutation at a rate of *M*(0)(*t*). The cells with one mutation divide at a rate *G*(1)(*t*); at a rate *D*(1)(*t*) they die or differentiate. Each cell with one mutation can also divide into an equivalent daughter cell and another cell with a second irreversible mutation at a rate *M*(1)(*t*). For the cells with two mutations there are also assumed to be competing processes of cell growth, differentiation, and mutation taking place at rates *G*(2)(*t*), *D*(2)(*t*), and *M*(2)(*t*), respectively, and so on until at the (*k*-1)th stage the cells which have accumulated (*k*-1)mutations proceed at a rate *M*(*k*-1)(*t*) to acquire another mutation and become malignant. The model is illustrated schematically in Figure 5. The two-mutation model of Moolgavkar, Venzon, and Knudson corresponds to the case *k *= 2. The classical Armitage-Doll multi-stage model corresponds to the case in which the intermediate cell proliferation rates *G*(*i*)(*t*) and the cell differentiation rates *D*(*i*)(*t*) are all zero.

It can be shown [4] that the excess risk for either model following a perturbation of the parameters will tend to zero as the attained age tends to infinity. One can also demonstrate that perturbation of the parameters *M*(*k*-2), *M*(*k*-1), *G*(*k*-1), and *D*(*k*-1) will result in an almost instantaneous change in the cancer rate [4]. In particular, this demonstrates that only models with *k ≥ *3 cancer stages have parameters that can be altered without instantaneous modification of the cancer hazard.

Generalized MVK models have been fitted to a number of datasets, in particular the Japanese atomic bomb survivor LSS Report 11 mortality data [123,126] and the Colorado Plateau uranium miners [117], as well as a group of radon-exposed rats [113], and give good fit, with in all cases the three-mutation model fitting at least as well as, and in some cases better than [117] (see also Figure 4), a model with two mutations. Little *et al*. [127] also showed that the age-incidence relationship for lymphocytic leukaemia incidence in the UK population could be adequately described by models with either two or three stages.

### Multiple pathway models

Little *et al*. [128] fitted a generalization of the Armitage-Doll model to the Japanese atomic bomb survivor and IRSCC leukaemia data which allowed for two cell populations at birth, one consisting of normal stem cells carrying no mutations, the second a population of cells each of which has been subject to a single mutation. The leukaemia risk predicted by such a model is equivalent to that resulting from a model with two pathways between the normal stem cell compartment and the final compartment of malignant cells, the second pathway having one fewer stage than the first. This model fitted the Japanese and International Radiation Study of Cervical Cancer Patients leukaemia datasets significantly better, albeit with biologically implausible parameters (the number of initiated cells at birth is negative), than a model which assumed just a single pathway [128]. A number of other such models are described by Tan [108] and Tan *et al*. [129], who also discuss at some length the biological and epidemiological evidence for such models of carcinogenesis.

We now discuss what may appear to be a special case of these multiple pathway models, but which are of sufficient flexibility to embrace most categories of multiple pathway models.

### Multiple pathway models incorporating genomic instability

As discussed in the section "Genomic instability and somatic cellular Darwinian evolution in cancer" there is biological data suggesting that the initiating lesion in the multistage process leading to cancer might be one involving a destabilization of the genome resulting in elevation of mutation rates [68,69]. There have been a few attempts to incorporate GI in mechanistic carcinogenesis models [130,131], although in general these models have not been fitted to data in a statistically rigorous manner. Little and Wright [5] developed a stochastic carcinogenesis model which allowed for genome destabilization, very close in spirit to the model of Mao *et al*. [130], and generalizing the class of generalized MVK models developed by Little [4,123,126], which in turn therefore generalize the two-mutation model of Moolgavkar, Venzon and Knudson [2,3]. Little *et al*. [6] developed a generalization of the model of Little and Wright [5] that allowed for multiple types of GI, and have fitted the model to SEER population-based Caucasian colon cancer incidence data.

The more general model of Little *et al*. [6] makes the following assumptions:

1. Malignancy arises from a series of genetic transformations of a stem cell;

2. Cells can undergo two classes of mutations, cancer-stage mutations or destabilizing mutations. Both are irreversible;

3. Multiple types of GI can occur, which are mutually exclusive - once cells are committed to a particular type of GI they and their daughter cells cannot exhibit any other type of GI;

4. Conditional on their ancestry and model parameter history to date, at any stage of the cancer process, cells are statistically independent of each other;

5. A tumour cell that has experienced the required number of cancer mutations will develop into a clinically detectable tumour.

**Figure 6:**
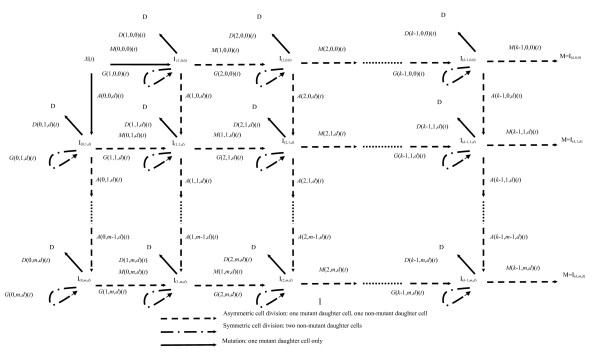
**Schematic diagram of generalized cancer model with *k *cancer-stage mutations and *m *destabilizing mutations, as in Little *et al*. **[6]. This corresponds to a single type, *d*, destabilizing mutation (*d *∈ [1, *r*]) with *m *= *m*_*d *_destabilizing levels. When there is more than one type of destabilizing mutation, there are multiple copies of this diagram, glued together along the topmost axis (of cells that have not acquired a destabilizing mutation), as in Figure 7.

**Figure 7:**
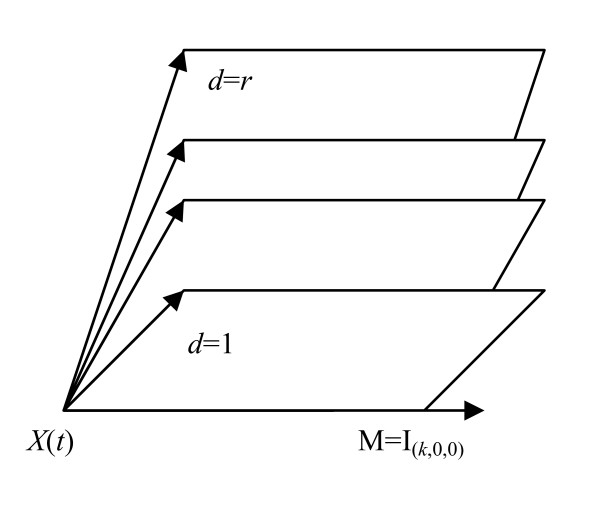
**Schematic diagram of the various destabilizing mutation planes in the model of Little *et al*. **[6], **each plane with the structure of Figure 6**. Under the assumption of mutually exclusive destabilizing mutations, cells that have committed to one type of GI are not allowed to move between these planes.

Cells can acquire up to *k *successive cancer-stage mutations, and any of *r *(mutually exclusive) types of destabilization mutation(s), e.g., of CIN or MIN type. Cells become malignant when *k *cancer-stage mutations have occurred, no matter how many destabilizing mutations there have been. Once a cell has acquired a destabilizing mutation of type *d *(1 ≤ *d ≤ r*), it and its daughter cells can acquire up to *m*_*d *_- 1 further destabilizing mutations of the same type. We define *r *to be the ***multiplicity of destabilization mutation types***. It is to be expected that the more destabilizing mutations cells acquire of each type, the higher the cancer stage mutation rate is, but this is not intrinsic to the model. The assumption that the *r *destabilization types are mutually exclusive is known to be the case for CIN and MIN in relation to colon and endometrial cancer [27]. The model is illustrated schematically in Figures 6 and 7.

**Figure 8:**
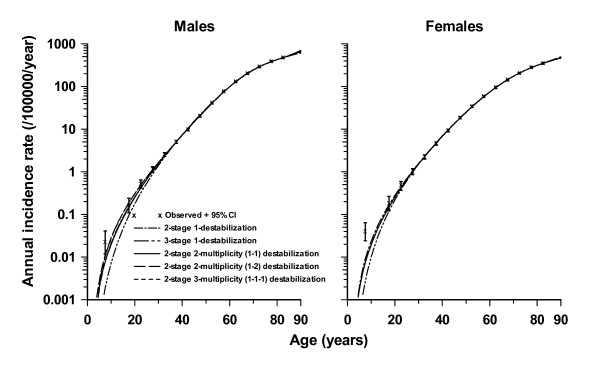
**Observed colon cancer rate (and 95% CI, adjusted for overdispersion) and model predicted rates for the Caucasian male and female population, taken from Little *et al*. **[6]. Rates are those predicted by the (single multiplicity) models with two cancer-stage mutations and one destabilizing mutation and three cancer-stage mutations and one destabilizing mutation. Also shown are the predicted rates for the models with two cancer-stage mutations with multiplicity two and (1-1) destabilizing mutations (i.e. 2-2-(1-1)), with multiplicity two and (1-2) destabilizing mutations (i.e. 2-2-(1-2)) and with multiplicity three and (1-1-1) destabilizing mutations (i.e. 2-3-(1-1-1)). The stem cell population is fixed at 10^8 ^cells [166].

Cells at different stages of the process are labelled by *I*_(*α, β, d*)_, where the first subscript, *α*, represents the number of cancer stage mutations that the cell has accumulated, the second subscript, *β*, represents the number of destabilizing mutations acquired, their type being given by the third subscript, *d*. At all stages other than *I*_(0,0,0)_, cells are allowed to divide symmetrically or differentiate (or undergo apoptosis) at rates *G*(*α, β, d*) and *D*(*α, β, d*), respectively. Each cell can divide into an equivalent daughter cell and another cell with an extra cancer stage mutation at rate *M*(*α, β, d*). Likewise, cells can also divide into an equivalent daughter cell and another cell with an additional destabilizing mutation of type *d *at rate *A*(*α, β, d*). The model assumes that there are *X*(*t*) susceptible stem cells at age *t*. The acquisition of carcinogenic (cancer-stage) mutations amounts to moving horizontally (left to right) in Figure 6, whereas acquisition of destabilizing mutations amounts to moving vertically (top to bottom) in this figure. Further mathematical details on derivation of the hazard function for this model are given in Appendix A. The two-mutation MVK model corresponds to the case *k *= 2, *r *= 1, *m *= *m*_1 _= 0, while the generalized MVK model with *K *stages developed by Little [4,123,126] amounts to the case *k *= *K*, *r *= 1, *m *= *m*_1 _= 0. However, in fits to the SEER colon cancer data, there is little evidence to support the hypothesis that the model with more than one type of genomic instability fits better than models with a single type of genomic instability [6] (see Figure 8), nor is there evidence that these models fit the data any better than a model (similar to that used by Luebeck and Moolgavkar [91]) that did not assume GI [89]. However, Tan and Tan [132] fitted very similar multiple pathway models to virtually the same SEER data and found stronger evidence for the involvement of genomic instability. The reasons for the somewhat different conclusions from our own probably relate to the incorporation of more biological data (via highly informative priors) by Tan and Tan [132], achieved using Bayesian model fitting techniques.

It is vital in fitting these and other models to take account of problems of parameter identifiability. It has been known for some time that there is redundancy in the parameterization of the two-mutation model, so that only three combinations of the five available combinations of model parameters (*X*, *M*(0), *M*(1), *G*(1), *D*(1)) can be estimated from knowledge of the hazard function [133-135], i.e., two combinations of parameters cannot be estimated. There is a large literature on this, the most important parts of which can be found in the articles of Heidenreich *et al*. [136] and Hanin [135]. More general material on parameter identifiability and redundancy can be found in the papers by Rothenberg [137], Jacquez and Perry [138], Catchpole and Morgan [139] and Little *et al*. [140]. Little *et al*. [141] have extended the results of Heidenreich [134] and Heidenreich *et al*. [136], showing that for the class of models considered by Little and Wright [5], that includes the two-mutation model as a special case, two parameter combinations cannot be estimated; more generally, for models of the sort constructed by Little *et al*. [6] with *r *types of destabilization, there are at least *r *+ 1 parameter redundancies, i.e., the number of estimable parameters is no more than the number of biological parameters minus *r *+ 1 [141].

### Malignant cell growth and clonal extinction

The models discussed above deal with the generally prolonged multistage process whereby a cell and its offspring successively accumulate mutations which result in the production of a cell with a malignant phenotype. What is usually not modelled is the final (and relatively short) stage in tumour development, from the appearance of the first malignant cell up to the clinically overt tumour; this is usually set to some constant (e.g., [5,6,114]). However, as noted above, the generalized multistage models of Little [4], Little and Wright [5] and Little *et al*. [6] allow for modelling of a final stochastic-growth or stochastic birth-death process of tumour growth from the first malignant cell; in particular this last process could be used to model the "angiogenic switch".

There is a large literature on models of tumour growth and angiogenesis from the appearance of the first malignant cell, the most recent parts of which we now briefly review. Basanta *et al*. [142] use evolutionary game theory to model glycolysis and its role in tumour invasion and progression. Komarova *et al*. [143] utilize a system of logistic ordinary differential equations (ODE) to model the total and mutant cell population, in which mutants are generated by one-stage oncogene activation and two-stage TSG inactivation. D'Onofrio and Gandolfi [144] model tumour and vascular growth using ODEs, as also do Ledzewicz and Schättler [145], using also ideas from optimal control theory. Enderling *et al*. [146] employ an agent-based approach to model tumour growth, migration and cell death; a similar approach is adopted by Wcisło *et al*. [147], who also modelled vascular growth. Macklin *et al*. [148] use solutions of reaction-diffusion partial differential equations (PDE) to spatially model tumour growth and migration and nutrient supply; a similar approach is adopted by Anderson [149]. Gatenby *et al*. [55] present compelling evidence that, at least for breast cancer, there is late-stage somatic evolution of epithelial cancer cells entirely within the space contained by the basement membrane. Gatenby *et al*. [55] propose a mathematical model that allows for somatic evolution in development of breast cancer resulting in up-regulation of glycolysis to maintain ATP production despite hypoxia, as well as mutations to reduce acid-mediated toxicity. Smallbone *et al*. [56] develop these ideas and construct a schematic model that suggests that transient exercise-induced acidosis may be sufficient to disrupt these critical somatic mutations; this may mediate the observed reduction of cancer risk with exercise. A problem in all of these papers is that no attempt has been made to fit the models to biological or clinical data, and model parameters appear to have been chosen aribitrarily. Slightly older literature in this area is reviewed in the text of Adam and Bellomo [150].

### Cell cycle models

The models discussed above inevitably leave out much biology. One aspect of cancer and normal cell biology that may be of importance is the cell cycle, because the cell-cycle checkpoint machinery is critical for DNA damage and repair, reviewed above, also because of the known variation of cellular radiosensitivity with cell-cycle stage [151-153]. Alarcón *et al*. [154] performed simulations of the cell cycle in normal and cancer cells via a system of ODEs. Hazelton [155] outlined simulations using a similar ODE system integrated within a model of carcinogenesis. A slightly more complex model is that of Ribba *et al*. [156], a spatial model of cell-cycle and cell migration, simulations from which were employed to assess regulation of tumour growth subject to radiotherapy. None of these models appear to have been rigorously fitted to data.

## Discussion

All mathematical models make assumptions; these assumptions simplify the underlying biology, and are often made for reasons of mathematical or statistical tractability. We have discussed some of these here, in particular the critical assumption of somatic cellular Darwinian evolution, or conditional independence of transformed cell populations, which we think may be justified. However, one would be wise to admit that there is still a lot that is not known about the cancer process, and to this extent a degree of caution is advised in using these models.

For example, it is not altogether clear that the assumption we make that cells can only acquire a single sort of destabilization is correct. This assumption is made to simplify the mathematics and is based upon the inverse relationship observed in colorectal cancer [27]. Human colorectal cancer cells that exhibit CIN do not have alterations in the MMR genes whereas cells with defective MMR mechanism are near diploid and do not manifest abnormalities associated with CIN [27]. Moreover, the genetic alterations in CIN and MIN cells are generally distinct. CIN related cell lines have mutations in *p53 *and *APC *[157]. In contrast, MIN cells have frameshift mutations in genes such as *β*-catenin and *TGF-β RII *[158,159], but seldom display *p53* and *K-ras *mutations [160]. Cell fusion studies also provide insight into the relationship between CIN and MIN. Lengauer *et al*. [75] demonstrated in a cell fusion experiment that wild-type MMR genes in CIN cells restored MMR function in MIN cells, resulting in the expression of CIN but not MIN in a hybrid population of the two cell types.

As noted in the sub-section "Multiple pathway models incorporating genomic instability", there is little evidence to indicate that models with GI, let alone models that assume multiple types of GI, yield better fit than models that do not assume GI [6,89] although conclusions at variance with this have been reached by other modelling groups [132]. One reason could be that data containing information only on the age distribution of cancer does not possess the power to discriminate between models and hence to confirm or to falsify the hypothesized involvement of GI in colon cancer. Given how well some of these simpler models fit this data (e.g., the two cancer-stage one destabilization (2-1) model), it is perhaps unremarkable that Little *et al*. [6] do not find much improvement in fit offered by the models that allow for multiple types of GI. It should be noted that Little *et al*. [6] are concerned mainly with relative goodness of fit, as determined, for example, by use of likelihood ratio tests. Further investigation of minor variant models by Little *et al*. [6] did not suggest marked modifications to these conclusions. These considerations are also supported by Hornsby *et al*. [161], who showed that modest changes in model specification can be difficult to distinguish in their effect on the cancer incidence rate. Quantitative information on exposure to various mutagenic agents (e.g., ionizing radiation) would better discriminate between models, as would comparison of the age-specific incidence of inherited and non-inherited forms of cancer [3,162]. Knudson [3] examined incidence of inherited and sporadic forms of retinoblastoma and inferred that two mutations were responsible for inducing this type of tumour. Frank [162] fitted a simple multistage model, similar to that of Armitage and Doll [1], to data on retinoblastoma and colorectal cancer. By assuming the inherited form to have one rate-limiting stage less than its non-inherited counterpart, the ratio of the incidence of non-inherited and inherited forms could be used to discriminate between models [162]. The colon cancer data used by Little and Li [89] and Little *et al*. [6] lack information on heritability, but other datasets that have this information (e.g., [163]) could be used to facilitate discrimination between models.

## Abbreviations

**DNA**: deoxyribonucleic acid; **DSB**: double strand break; **GI**: genomic instability; **HNPCC**: hereditary non-polyposis colorectal cancer; **HR**: homologous recombination; **LOH**: loss of heterozygosity; **LSS**: Life Span Study; **MMR**: mismatch repair; **MVK**: Moolgavkar, Venzon, Knudson; **NHEJ**: non-homologous end joining; **ODE**: ordinary differential equation; **PDE**: partial differential equation; **RB**: retinoblastoma; **TSG**: tumour suppressor gene.

## Competing interests

This author declares that they have no competing interests.

## Authors' contributions

The author planned and wrote the paper.

## Reviewers' comments

### Comments from Reviewer 1 (RA Gatenby)

A very nice and thorough review. I would like to suggest that you also consider the role of the unique tumor environment since Darwinian dynamics consists of both heritable changes and environmental selection forces which can be both spatial and temporally heterogeneous. Cancers evolve on epithelial surfaces and are separated from their blood supply by an intact basement membrane. This creates very specific environmental selection forces and different stages of premalignant tumor growth. This allows the specific mutations observed in cancer to be understood as adaptations to these microenvironmental factors.

### Response to Reviewer 1

Agreed. This is a good point. I have added some extra sentences in the sub-section "Malignant cell growth and clonal extinction" making very much these points. I also refer to these ideas briefly at the start of the section "Genomic instability and somatic cellular Darwinian evolution in cancer".

### Comments from Reviewer 2 (M Kimmel)

Recently, there has been a surge in interest in the cancerization field theory of carcinogenesis, which states that as a result of exposure to carcinogens and/or of inherited genetic variants (mutations), a substantial portion of an organ (called the field) can be enriched in genetic variants of cells, which then may or may not acquire further genomic modifications. Cells in the field may or may not be clonal. The modifications will result in increased proliferation and invasion of the surrounding tissues.

Because of the spatial dimensions of the field, emerging groups of transformed cells (precancerous and early cancerous tumours) will represent different levels of transformation, and may exhibit both progression. They will be frequently multifocal. This viewpoint is in opposition to the clonal theory of carcinogenesis, which implies linear irreversible progression and generally unifocal lesions. Assuming that the field theory is true, the models of early cancer growth will have to be revised. What impact, will this have on models presented in the current paper?

### Response to Reviewer 2

This topic is considered in para. 2 of the section "Genomic instability and somatic cellular Darwinian evolution in cancer". I do not judge that the field theory is necessarily in contradiction with the idea of cancer as a failure of diferentiation. However, I do not think that it can account for the initiation (rather than progression) of most tumours, since it demonstrably fails to account for the clonality that is observed in many cancers, as I point out in this section.

## Appendix A. Details of hazard function derivation for the model of Little et al. [6]

Let *Y*_*α, β, d *_(*t*) denote the number of cells with *α *cancer stage mutations, *β *destabilizing mutations of type *d *at time *t*, and *Y*_*k*_(*t*) denote the number of malignant cells (cells that have acquired *k *cancer stage mutations). Let us define the full probability generating function (PGF):

Let *ϕ *be the corresponding partial probability generating functions,

which starts with 1 cell in compartment *I*_(*α, β, d *)_at time *s *and with no transitions into that cell from cells *I*_(*α', β', d*) _with *α*' <*α *or *β*' <*β*. Notice that *ϕ*_*k, β, d *_[*t,s*] = *y*_*k*_. The partial PGFs satisfy the Kolmogorov forward equations, given by:(A1)

where 0 ≤ *α *≤ *k *-1, 0 ≤ *β *≤ *m*_*d*_, 0 ≤ *d *≤ *r*, (*α, β, d*) ≠ (0,0,0), 1_*d *= 0 _is the indicator function defined by  and similarly . We adopt the convention that *y*_*k*, *β*, *d *_≡ *y*_*k *_and  for any *α, β, d*, and that *A*(*α, β, d*) = 0 for *β *≥ *m*_*d*_. Similarly, the Kolmogorov backward equations for *ϕ*_*α, β, d *_[*t, s*] are given by(A2)

with the same range for each *α*, *β *and *d*. We adopt the convention that . The hazard function, *h*(*t*), is the probability that the appearance of the first tumour cell is at time *t*, defined by:

where *T *is the time that a malignant cell develops for the first time. As in Little and Wright [5] we can easily derive:

Thus *h*(*t*) can be written as:(A3)

In order to calculate the hazard function, we differentiate the backward equations (A2) with respect to *t *and obtain the following equations:(A4)

for 0 ≤ *α *≤ *k*-1, 0 ≤ *β *≤ *m*_*d*_, 0 ≤ *d *≤ *r *and (*α, β, d*) ≠ (0,0,0).

### Boundary conditions

From the forward equations (A1), we can obtain the boundary conditions for :(A5)

By definition, the *ϕ*'s satisfy the boundary conditions given by:(A6)

### Procedures for calculating the hazard function

1. Using the Kolmogorov backward equations (A2) and their derivatives (A4), regarded, for fixed *t *as a set of ordinary differential equations (with respect to *s*) in the vector quantity  together with the boundary conditions (A5) and (A6), we obtain the solutions for *ϕ*_*α, β, d *_[1, 1,..., 1, 0; t, s] and  for all *α, β *and *γ *except (*α, β, d*) = (0,0,0).

2. By means of the mathematical trick outlined by Little and Wright [5], with little extra work this set of equations can be augmented to yield the hazard function and the cumulative hazard function. Let us write:(A7)

Then by (A3) *h*(*t*) = *g*(*t*, *s*)|_*s *= 0 _and *g*(*t*, *s*) satisfies:(A8)

3. Now define , so that . Then it is readily verified that:

with the initial condition *k*(*t*, *t*) = 0. Therefore, by augmenting the sets of differential equations (A2) and (A4) with equations (A8) and (A9) we derive the hazard function and its integral as desired.
